# Ruptured Ovarian Cyst Leading to Massive Spontaneous Intraperitoneal Hemorrhage in Chronic Immune Thrombocytopenic Purpura (ITP): Successful Nonsurgical Intervention

**DOI:** 10.7759/cureus.66979

**Published:** 2024-08-16

**Authors:** Rinkle Gemnani, Shilpa A Gaidhane, Pallavi Yelne, Ajinkya Kadu, Vineet Karwa

**Affiliations:** 1 Department of Medicine, Jawaharlal Nehru Medical College, Datta Meghe Institute of Higher Education and Research, Wardha, IND

**Keywords:** case report, ruptured ovarian cyst, hemoperitoneum, corpus luteum hemorrhage, immune thrombocytopenia

## Abstract

Immune thrombocytopenic purpura (ITP) patients typically exhibit mild mucocutaneous bleeding. Although corpus luteum hemorrhage (CLH) is usually asymptomatic, it can rarely result in serious intraperitoneal bleeding, usually in patients with coagulation abnormalities. In cases of severe thrombocytopenia, hemoperitoneum during ovulation during an ITP flare-up is an extremely unusual and potentially fatal event. Our patient, a 25-year-old multiparous female with a known case of ITP, presented with a spontaneous petechial rash all over the body, abdominal pain, and dizzy spells. Hemoglobin was 6.3 g/L with a platelet count of 0.04 × 10^9^/L and a negative urine pregnancy test. An abdominopelvic ultrasound revealed free fluid in the peritoneal cavity, and a contrast-enhanced computed tomography (CECT) confirmed hemoperitoneum with a ruptured ovarian cyst. We report the case of a young female with ITP relapse who landed into massive intraperitoneal hemorrhage and was managed conservatively with platelet transfusion, steroids, and romiplostim.

## Introduction

Immune thrombocytopenic purpura (ITP) is a hematological disorder characterized by a platelet count of less than 100 × 10^9^/L due to autoimmune platelet destruction and decreased platelet production [1,2]. The disease is benign and chronic, affecting people of all ages. The mean age of diagnosis is between 20 and 50 years, and the ratio of females to males is over 3:1 [2]. This illness usually presents in either an acute or chronic form. ITP typically shows no symptoms, with symptoms only manifesting at extremely low platelet count levels [1]. The typical spectrum of symptoms includes mild bleeding presentations like ecchymoses, purpura, and petechiae, which usually affect the extremities, as well as serious bleeding that necessitates hospitalization, such as epistaxis, cerebral hemorrhage, gastrointestinal bleeding, and so forth [2].

A transient endocrine structure called the corpus luteum is created when the follicle luteinizes following ovulation. Various factors, such as coitus, trauma, exercise, or vaginal inspection, might induce or provoke corpus luteum bleeding. Consecutively, hemostatic illnesses, including ITP and hemophilia, may enhance the risk of hemorrhagic rupture of the corpus luteum [3]. While hemorrhagic ovarian cysts are not unusual, they can occasionally rupture and cause spontaneous acute hemoperitoneum, which can be fatal in the event of severe thrombocytopenia. We present the case of a young Asian woman who was diagnosed with ITP and is currently in acute relapse. She developed a major hemoperitoneum as a consequence of an ovulation-related coincidental corpus luteal rupture.

## Case presentation

A 25-year-old married multiparous female presented to our emergency room with complaints of abdominal pain, purpuric patches over her hands, trunk, and lower extremities, and dizziness for one day. Initially, the abdominal pain started in the left lower abdomen and was localized, which gradually progressed in severity and became generalized over 12-14 hours. The patient also has a history of four to five dizzy spells in the past day. Her last menstrual period was 11 days ago, without any previous history of abnormal uterine bleeding. The patient has had a known case of chronic ITP for two years and has been on an injection of romiplostim (4 µg/kg) subcutaneously weekly for the last two months. There is a history of multiple blood transfusions in the past two years.

**Figure 1:**
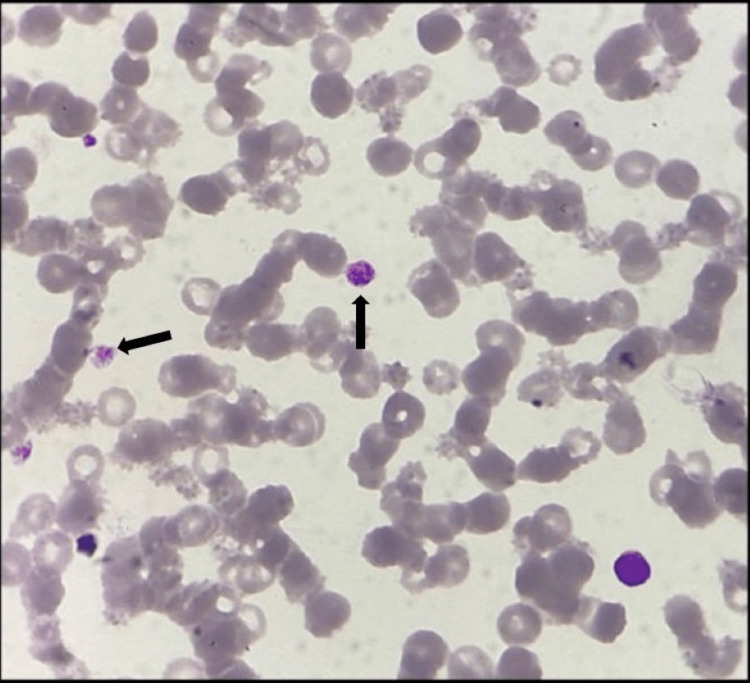
Reduced platelet count in peripheral smear (black arrow).

On arrival, the patient was conscious and oriented. She looked pale and apprehensive, with cold and clammy skin. Her pulse rate was 110 beats per minute, and her blood pressure was 80 mmHg systolic. Her oxygen saturation was 98% on room air. On examination, she had severe pallor and generalized petechial hemorrhage on her face, trunk, and extremities. On abdominal palpation, guarding and tenderness were present over the epigastric and left lumbar regions. Her complete blood count revealed a hemoglobin of 6.3 g/dL, a platelet count of 0.04 × 10^9^/L (Figure 1), and a total white blood cell count of 20,300 cells/cumm with a normal prothrombin time, activated partial thromboplastin time, and international normalized ratio.

Other laboratory parameters are given in Table 1.

**Table 1:** Laboratory parameters of the patient with reference ranges

Lab parameters	Observed value	Normal range
Hemoglobin	6.3 g/dL (low)	12-15 g/dL
Mean corpuscular volume	79 fL (low)	83-101 fL
Total leukocyte count	20,300 cells/cumm (high)	4,000-10,000 cells/cumm
Platelets	0.04 lakhs/cumm (low)	1.5-4.1 lakhs/cumm
Activated partial thromboplastin time	29.9	29.5-31
Prothrombin time	13	11.9-13
International normalized ratio	1.01	0.9-1.4
Serum urea	27 mg/dL	19-43 mg/dL
Serum creatinine	0.6 mg/dL	0.66-1.25 mg/dL
Serum sodium	141 mmol/L	137-145 mmol/L
Serum potassium	4.0 mmol/L	3.5-5.1 mmol/L
Alkaline phosphatase	81 U/L	38-126 U/L
Alanine aminotransferase	32 U/L	<50 U/L
Aspartate aminotransferase	38 U/L	17-59 U/L
Albumin	3.8 g/dL	3.5-5 g/dL
Total bilirubin	0.8 mg/dL	0.2-1.3 mg/dL
Erythrocyte sedimentation rate	10 mm/hour	0-20 mm/hour

There were no previous documents available to compare. An ultrasound of the abdomen was done, which showed a massive hemoperitoneum with a left-sided hemorrhagic ovarian cyst, and a diagnosis of ruptured ectopic was considered. Subsequently, a urine pregnancy test was done, which was negative, and beta-human chorionic gonadotropin hormone was 2.39 mIU/mL (>25 mIU/mL is positive); hence, a contrast-enhanced computed tomography (CECT) of the abdomen was planned.

**Figure 2:**
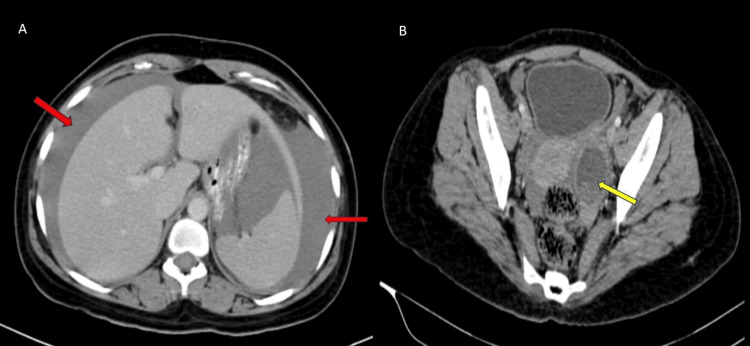
CECT of the abdomen and pelvis showing (A) hypodense fluid in the abdomen and pelvis suggestive of hemoperitoneum (red arrow) and (B) well-defined peripherally enhancing cystic lesion in the left ovary with the hyperdense non-enhancing component within the cyst suggestive of the hemorrhagic cyst (yellow arrow). CECT, contrast-enhanced computed tomography

CECT of the abdomen revealed evidence of a well-defined peripherally enhancing cystic lesion in the left ovary measuring 4.3 × 3.5 × 5.1 cm with a hyperdense non-enhancing component within the cyst suggestive of hemorrhagic cyst with moderate hyperdense fluid in the abdomen and pelvis suggestive of hemoperitoneum (Figure 2).

After the hematologist's opinion, the patient was started on blood transfusion (total of two whole blood), random donor platelet (RDP) transfusion (total of 16 RDPs), steroid (injection methylprednisolone 1 g IV stat followed by 500 g IV OD for three days), and injection romiplostim 250 mcg subcutaneous single dose along with injection leuprolide acetate 3.75 mg intramuscularly (IM) stat. In view of the massive intraperitoneal hemorrhage and unstable general condition of the patient, an exploratory laparotomy was planned, but even after the transfusion of 10 RDPs in 48 hours, the patient's platelet count was 0.18 × 10^9^/L. Hence, the surgery plan was delayed. The patient was continuously monitored for vitals like pulse rate, blood pressure, and oxygen saturation. Over the next 48 hours, the patient showed a dramatic improvement in her symptoms. Her abdominal girth was persistently decreasing; there were no signs of active bleeding, and the patient became vitally stable; hence, conservative management was considered. The platelet count rose to 1.62 × 10^9^/L over the next six days, as shown in Table 2, and the patient was discharged.

**Table 2:** The number of RDPs transfused during the hospital stay and platelet counts after transfusion RDP, random donor platelet

Days	No. of RDPs transfused	Total platelet count
On admission	4	0.04 × 10^9^/L to 0.10 × 10^9^/L
Second day	6	0.18 × 10^9^/L
Third day	6	0.30 × 10^9^/L
Fourth day	0	0.67 × 10^9^/L
Fifth day	0	1.29 × 10^9^/L
Sixth day	0	1.62 × 10^9^/L

A follow-up ultrasound after seven days revealed a resolving hemoperitoneum with a left hemorrhagic cyst measuring 2.1 × 1.6 cm and platelet counts of 2.0 × 10^9^/L. In our case, the patient was successfully managed conservatively with steroids and romiplostim. The patient was advised of splenectomy but was not willing.

## Discussion

As per the etiological classification, ITP can have primary and secondary causes, of which the primary ITP is the diagnosis of exclusion [4]. The etiology is generally considered to be an immune-mediated process, though it is idiopathic in certain cases. A prior viral infection, drugs (e.g., NSAIDs, penicillin, quinine), and malignant conditions like leukemia are examples of secondary causes [1]. In ITP, platelet counts below 50 × 10^9^/L are associated with an increased risk of traumatic bleeding, while values below 20 × 10^9^/L are associated with an increased risk of spontaneous bleeding [5]. Mucocutaneous symptoms, such as petechial rash and mucosal membrane bleeding, are typical of its presentation. Menorrhagia and epistaxis are possible additional symptoms; more serious ones could involve intracerebral hemorrhage or gastrointestinal bleeding [5].

An ovum develops inside the follicle and is discharged from the ovarian surface into the peritoneal cavity during ovulation. This phase is associated with the luteal phase in the menstrual cycle. In the absence of pregnancy, the corpus luteum shrinks, becomes a functional cyst with thin walls, and is extremely vascular, which makes it more vulnerable to bleeding [6]. Bleeding from the ruptured follicle or the luteal cyst in the presence of a bleeding diathesis, such as ITP, may pose an increased risk of spontaneous hemoperitoneum.

The bleeding might be limited to the cyst or may extend to the peritoneal cavity. The rupture of an ectopic pregnancy or a hemorrhagic ovarian cyst is two crucial differentials to rule out in a woman of reproductive age who presents with progressive hypotension and syncopal episodes. A urine pregnancy test in our situation came out negative. Hence, a ruptured ovarian cyst had a high index of suspicion, which was confirmed by a CECT. Hallatt et al. provided the first noteworthy series of individuals with hemoperitoneum with corpus luteum hemorrhage when they characterized this phenomenon. They observed varying quantities of hemoperitoneum at the moment of exploration and acknowledged that this event may occur at any point in the reproductive life cycle [7]. There have been few rare case reports published linking corpus luteum hemorrhage to spontaneous hemoperitoneum in patients with bleeding disorders, including women with aplastic anemia, ITP, hemophilia or hemophilia carrier status, afibrinogenemia, von Willebrand disease, and factors X, VII, V, II, and XIII deficiency [1,8]. Nevertheless, it is not so common for a woman with ITP to suffer from substantial bleeding, which is life-threatening.

Treatment must commence when the platelet count falls to the point where there is a risk of severe bleeding. The decision to treat these patients should be based on the severity of bleeding, drug adverse effects, and health-related quality of life [9,10]. In individuals with a ruptured ovarian cyst and hemoperitoneum who have no bleeding diathesis, a conservative approach is usually the mainstay of treatment, with continuous vital signs monitoring, platelet and hematocrit levels, and reassessment of the patient [1]. Unstable vitals observations, a drop in hemoglobin levels, an increase in hemoperitoneum levels seen on repeat imaging, or persistent abdominal pain that is not relieved with analgesics are all indications for surgical intervention [11]. Radiology revealed a significant quantity of hemoperitoneum in our patient, who was symptomatic, hypotensive, and had a low hemoglobin level. Despite these conditions, the patient was successfully treated with conservative measures and was advised for splenectomy, but she refused. In summary, we have reported a rare case of spontaneous hemoperitoneum caused by a burst luteal cyst in the setting of an acute relapse of ITP. A comprehensive approach to management is necessary in such complex cases.

## Conclusions

We reported this rare case to underscore the importance of timely management of a massive hemoperitoneum, which could be life-threatening in the background of an ITP relapse. In this potentially fatal incident, a high index of suspicion, timely diagnosis, and early initiation of therapy can be beneficial. Laparotomy is not essential in all such circumstances, and judgments should be made for individual patients. In cases where the patient's laboratory results and overall health do not support an emergent laparotomy, intravenous corticosteroid therapy and platelet transfusions should be considered as life-saving measures.
